# Protective Effect of Asiaticoside on Radiation-induced Proliferation Inhibition and DNA Damage of Fibroblasts and Mice Death

**DOI:** 10.1515/biol-2020-0015

**Published:** 2020-04-06

**Authors:** Haiyan Shen, Fei Zhu, Jinsheng Li, Songjia Tang, Yale Zhang, Jufang Zhang

**Affiliations:** 1 Department of Plastic Surgery, Affiliated Hangzhou People’s Hospital Zhejiang University School of Medicine, Hangzhou, 310003, P.R. China

**Keywords:** Asiaticoside (AC), Radiation-induced injuries (RII), Antioxidant, DNA damage, Radio-protective

## Abstract

**Background:**

Radiation-induced injuries (RII) mainly result from reactive oxygen species (ROS), which are harmful compounds that can damage DNA. Asiaticoside (AC), one of the main functional components extracted from Centella asiatica, has potent pharmacological effects such as anti-inflammatory and anti-oxidant activity. However, its role in RII remains unclear.

**Purpose:**

The purpose of the current study is to investigate whether AC can mitigate RII *in vitro* and *in vivo*.

**Material and Methods:**

Cell model of RII was successfully established by 5J/m_2_ radiation *in vitro*. For the *in vivo* RII model, mice were irradiated with 5 Gy to the thorax. The degree of damage to cells or mouse tissue was determined by measuring the numbers of DNA double-strand breaks (DSBs), oxidative stress, and mouse survival rates.

**Results:**

In the *in vitro* assay, AC administration significantly reduced radiation-induced growth inhibition of Escherichia coli and fibroblasts, DSBs and apoptosis of fibroblasts; in the *in vivo* study, AC could decrease antioxidant capacity (T-AOC) of plasma and protect mice from RII, thereby improving the survival rates of mice after radiation.

**Conclusions:**

These novel data indicate that AC is able to prevent radiation-initiated genotoxicity by mitigating DNA damage, and might serve as a safe and effective radio-protective agent.

## 1 Introduction

Radiotherapy, one of the main methods that eradicates cancer of thoracic malignant tumors such as lung cancer, esophageal cancer and lymphoma, plays an important role in controlling the pernicious development of malignant tumor cells. However, along with the injurious effect on tumor tissues, radiation therapy induces injuries in normal tissues by the production of large amount of ROS and H_2_O_2_, which finally leads to radiation pneumonia and pulmonary fibrosis [1]. Amifostine, the only radio-protective medicine approved by FDA, has effective radio-protective ability. However, it also brings some negative side effects for the patients such as vomiting, hypertension and nausea [2]. Thus, it is urgent to develop a safe and effective therapeutic radio-protective agent for clinical use.

Radiation, as a non-specific stimulation, leads to a massive increase in ROS which subsequently attacks the DNA, resulting in functional damage of cells and tissues [3, 4]. Therefore, it is necessary to mitigate ROS to protect against radiation-induced injury. AC, a triterpenoid saponin, is one of the main functional components of Centella. Previous studies have shown that AC plays a positive role in anti-inflammation, anti-oxidation and promoting wound healing [5, 6, 7]. However, understanding of AC’s protective mechanism in radiation-induced injuries has been lacking.

In our study, we found that AC had a protective function to mitigate radiation-induced injuries in Escherichia coli and fibroblasts and fibrosis in mice, which provided new ideas for the development of agents to protect against radiation-induced DNA damage, cell apoptosis, cell fibrosis and provide an experimental basis for the use of AC as a radiation protection agent.

## 2 Materials and methods

### 2.1 Cell culture and drug treatment

Cells were cultured in Dulbecco’s modified eagle’s medium (DMEM) supplemented with 10% (v/v) fetal bovine serum (FBS). For drug treatment, sub-confluent cells were treated with 5 J/m_2_ radiation for 30 min followed by treatment with 400 μg/mL AC for indicated time. For the evaluation of survival rate, E.coli and fibroblasts were pretreated with 200 μg/mL AC before radiation.

### 2.2 MTT assay

6×10^4^ fibroblasts were seeded into 96-well plates according to the experimental grouping. After incubation for 48 h, 20 μL MTT solutions (5 g/L) was added to the cells for 4 h. 200 μL dimethyl sulfoxide was added to the cells after removing the supernatant and were vibrated for 10 min at low speed to make the crystals dissolve. Absorbance at the wavelength of 570 nm was read by microplate reader.

### 2.3 Animals

C57BL/6J mice (8-10 weeks old, male), which have been shown to be radiation sensitive, were housed five animals per cage under controlled conditions and received standard laboratory diet through the course of the experiment.

**Ethical approval**: The research related to animals use has been complied with all the relevant national regulations and institutional policies for the care and use of animals. Experiments were performed under protocols approved by the Committee on Animal Resources.

### 2.4 Irradiation treatment

The mice were randomly divided into four groups: Control, AC treatment, Radiation treatment, and AC plus Radiation treatment groups. For the irradiation, animals were restrained in plastic jigs and placed so that the thoraxes were located in the center of the field defined by the slit lead collimator and irradiated with 5 Gy to the thorax only. Treatment dose is based on the well-established threshold dose for the induction of fibrosis in the C57BL/6J strain, as previously reported using an identical exposure method. Animals were assigned to each treatment group for each time point examined. For AC treatment, AC-irradiated (5 Gy) mice received similar handling followed by administration of 25 mg/kg AC for 2 weeks on day 10 after radiation. After that, lung tissue of all mice was harvested and were used to detect histologic changes and protein/ mRNA levels of TGF-β1.

### 2.5 RT-PCR

RNA was extracted from frozen tissues with Trizol Reagent (Invitrogen) according to the manufacturer’s instructions. Two micrograms of RNA were reverse transcribed according to the manufacturer’s specifications. Multiplex PCR was done according to the previous description. Complementary DNA was made from RNA with a PrimeScript™ RT reagent kit, followed by quantitative PCR analysis according to the SYBR® Premix Ex Taq™ manufacturer’s instructions. The reaction program was set as follows: 37°C for 15 min, 95°C for 30 s for the initial template denaturation and 40 cycles of denaturation at 95°C for 5 s, followed by annealing and extension at 60°C for 30 s.

### 2.6 Comet assay

The comet assay was performed as previously described [8]. Briefly, gels were lysed overnight at 4°C. After lysis, gels were transferred to an electrophoresis chamber filled with alkaline unwinding buffer for 40 min at 4°C. Electrophoresis was conducted with the same buffer at 4°C for 30 min 1 V/cm and 300 mA. Neutralization solution was used to neutralize ethylene-bromide for 1min and then rinsed with deionized water to remove the excess dye. The number of comet tail cells was observed under the fluorescence microscope.

### 2.7 T-AOC assay

Total antioxidant capacity (T-AOC) in the serum of the mice were detected by a biochemical method following the instructions provided with the reagent kits [9] (T-AOC, A015; malondialdehyde, A003) purchased from Nanjing Jiancheng Bioengineering Institute of China.

### 2.8 Statistical analysis

Two-tailed Student’s t-tests for statistical analyses were performed with the data from three independent biological replicates using GraphPad Prism 5 to compare parameters between groups. Statistical significance was determined as p<0.05.

## 3 Results

### 3.1 AC increases cell viability of radiated or H_2_O_2_-treated Escherichia coli (E.coli)

**Figure 1:**
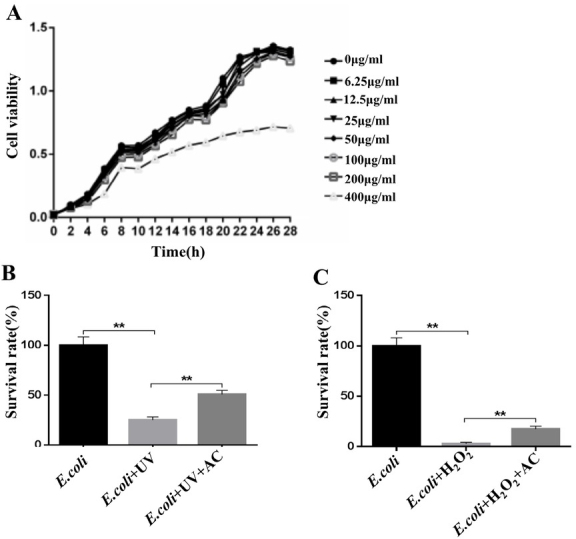
**Effect of AC on cell viability of radiation or H2O2-treated E.coli. (A)** The cytotoxicity of different concentrations of AC against E.coli.**(B)** The effect of AC on cell viability induced by 50 J/m^2^ doses radiation. **(C)** Variation of E.coli survival pretreated with 200 *μg*/mL AC with the treatment of H_2_O_2_. **, *P*<0.01.

It is confirmed that Escherichia coli lipopolysaccharide (LPS) can induce lung injury and release inflammatory factor NO [10]. In our study, 400 μg/mL AC inhibited proliferation and diminished the viability of E.coli, but there was no effect on the proliferation of E.coli when the concentration of AC was lower than 200 μg/mL **(Figure 1A)**. However, we found that pretreatment with 200 μg/mL AC before radiation significantly increased survival rates of E.coli as compared to that treated with radiation alone at 50 J/m^2^ doses **(Figure 1B)**. Similarly, AC administration increased cell viability of H_2_O_2_-treated E.coli **(Figure 1C)**. These data imply that the AC-mediated enhancement of E.coli cell viability might result in inhibition of LPS release, and subsequently the attenuation of the lung injury.

### 3.2 AC enhances cell viability of fibroblasts treated with radiation or H2O2

**Figure 2:**
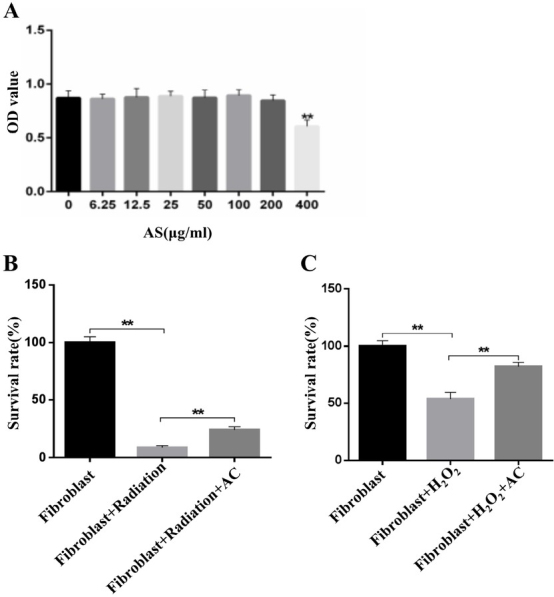
**Effect of AC on cell viability of radiation or H2O2-treated fibroblasts. (A)** The cytotoxicity of different concentrations of AC against fibroblasts. **(B)** The effect of AC on cell viability induced by 50 J/m^2^ doses radiation. **(C)** Variation of fibroblasts survival pretreated with 200 *μg*/mL AC with the treatment of H_2_O_2_. **, *P*<0.01.

Fibroblasts, the pivotal cell type in lung tissue, have a key role in protecting against lung injury. Dysfunction of fibroblasts leads to lung injury [11]. As shown in Figure 2A, AC reduced proliferation and diminished the viability of fibroblasts when the concentration of AC was more than 400 μg/mL, whereas lower than 200 μg/ mL did not affect cell viability of fibroblasts. However, pretreatment of fibroblasts with 200 μg/mL AC before radiation significantly promoted survival rates compared to fibroblasts treated with radiation alone at 6 Gy doses **(Figure 2B)**. Similarly, AC enhanced H_2_O_2_ treatment-mediated decline in fibroblast cell viability **(Figure 2C)**. These data suggest that AC attenuates cell apoptosis in irradiated fibroblasts.

### 3.3 AC attenuates DNA damage in radiated fibroblasts

**Figure 3:**
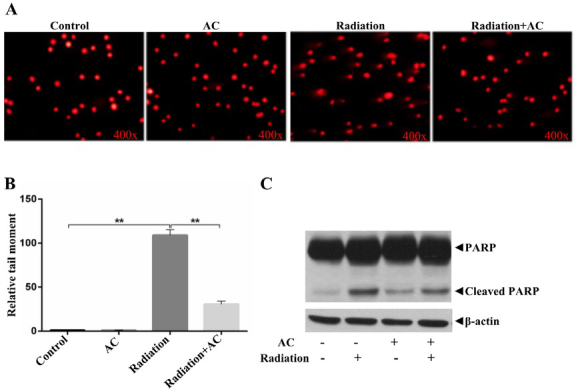
**Effect of AC on radiation-induced DNA damage in fibroblasts. (A)** Representative micrographs of comet assay. **(B)** Relative comet tail rate (%). **(C)** Protein expression of full-length PARP, Cleaved PARP and Actin by WB. **, *P*<0.01.

Next, we determined the effect of AC on DNA damage in fibroblasts. In our study, we discovered that irradiated fibroblasts resulted in an increase in the number of all comet tail moment, whereas pretreatment with AC before radiation clearly inhibited the increase of comet tail moment, indicating that AC had a protective effect on radiation-induced DNA damage **(Figure 3A and 3B)**. In addition, AC attenuated the apoptosis of irradiated fibroblasts with a decrease in cleaved PARP compared to radiation treatment alone **(Figure 3C)**.

### 3.4 AC improves survival rates of irradiated mice

**Figure 4:**
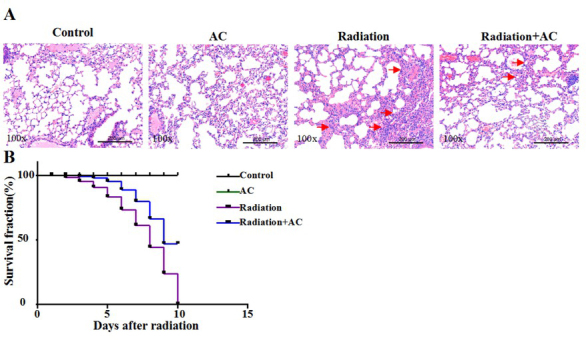
**Effect of AC on radiation-induced lung fibrosis and mice death. (A)** Histologic results of lung tissue in different group measured by HE staining. Scale bar: 200 μm. **(B)** Survival rates of radiated mice with or without AC treatment for 15 days.

We also evaluated the role of AC on radiation-evoked injuries *in vivo*. As shown in Figure 4A, lung tissue was severely damaged accompanied by increased fibrosis after radiation treatment. Once treated with AC, radiation-induced lung injury was significantly improved and the fibrosis level was obviously decreased **(Figure 4A)**. Consistent with the previous study, we also observed that all irradiated mice died on day 10 after irradiation alone, while 50% of the mice survived day 10 after radiation when treated with 25 mg/kg AC **(Figure 4B)**. These data show that AC improves the overall survival rates of irradiated mice.

### 3.5 AC increases the levels of plasma T-AOC and inhibits the expression of TGF-β1

**Figure 5:**
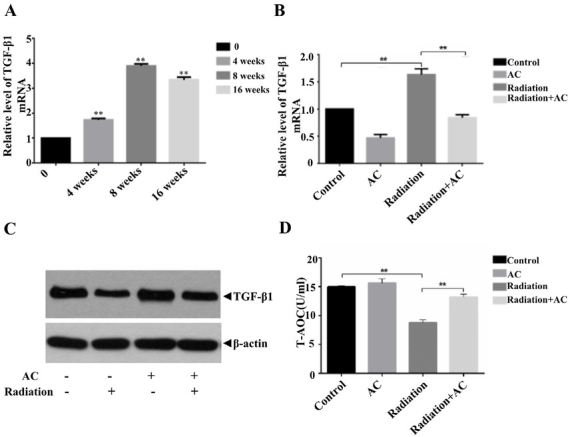
**Levels of Plasma T-AOC (a marker of oxidative stress) and the expression of TGF-β1. (A and B)** The level of TGF-β1 mRNA. **(C)** Observation of the expression of TGF-β1 by WB. **(D)** Levels of plasma T-AOC. **, *P*<0.01.

Radiation can induce lung injury and even pulmonary fibrosis. Elevated levels of TGF-β1 indicate the onset of pulmonary fibrosis [12]. Interestingly, we found that TGF-β1 mRNA was significantly increased with the passage of time under the condition of irradiation compared to the control, showing peak effect at 8 weeks which suggested that our *in vitro* model was successfully established **(Figure 5A)**. To explore the role of AC on radiation-mediated elevation of TGF-β1, we also chose 200 μg/mL as the treatment concentration. Like previous results, protein and mRNA levels of TGF-β1 in mice was robustly boosted after irradiation, whereas it was significantly reduced followed by the treatment of 25 mg/kg AC **(Figure 5B and 5C)**. Additionally, plasma T-AOC concentrations in the AC-treated group were significantly higher than those in the group treated with irradiation alone. Changes in the levels of T-AOC indicate the antioxidant potential of AC **(Figure 5D)**.

In summary, AC administration improves irradiation-mediated DNA damage and growth inhibition of fibroblasts. At the same time, AC enhances the level of T-AOC and inhibits the level of TGF-β1 in mice after irradiation, thereby prolonging survival. It appears that AC could serve as an effective and safe radio-protective agent.

## 4 Discussion

A side effect of radiation therapy is the generation of ROS that can result in oxidative deterioration of proteins and of DNA [13]. Elevated levels of ROS cause DNA damage, lung injuries and even pulmonary fibrosis [14]. It is known that antioxidants have an effective role in ameliorating radiation-induced oxidative injuries [15]. AC has been known to have anti-oxidant activity, however, the effect of AC on the progression of radiation-induced injury has not been studied [16]. In our study, we showed that AC increased the survival rates of irradiated or H_2_O_2_-treated cells, consistent with the previous study. AC attenuates radiation-induced injury, which may result from their radical-scavenging effect.

Previously, it has shown that acute exposure to radiation induces tissues injuries and mouse death [17]. Radiation also causes an increase in the expression of TGF-β1 to induce pulmonary fibrosis, resulting in cell apoptosis [18]. Similarly, the level of TGF-β1 in radiation-treated mice is significantly increased, and AC gavage significantly reduced the expression of TGF-β1 in mice. Continuous monitoring of radiation-treated mice for 15 days revealed that radiation caused a severe decline in survival rates of mice, and irradiated mice treated with AC significantly prolonged mouse survival, which indicates AC could be a potential radio-protective agent.

It is known that radiation induces DNA damage such as DNA double-strand breaks (DSBs) which are major cytotoxic lesions and pose a significant threat to genome stability if not properly repaired [19, 20]. Radiation induces large amounts of ROS targeting membrane lipids and DNA [21, 22]. DNA damage is one factor causing apoptosis of the cells [6, 23]. In our study, we found that AC significantly restored the level of serum T-AOC caused by radiation, and mitigated radiation-induced DNA damage, indicating that AC might act as a radio-protectant for the treatment of side effects produced by radiation therapy. Additionally, pretreatment of AC prior to radiation appeared to have a protective role against cell apoptosis and mouse death. Thus, the beneficial role of AC in radiation-induced damage is involvement with the cellular process of DNA damage and cell apoptosis.

In conclusion, AC has a radio-protective effect *in vitro* and *in vivo* possibly through its anti-oxidant power. However, the exact mechanism remains to be revealed in future.
